# SATB1 overexpression correlates with gastrointestinal neoplasms invasion and metastasis: a meta-analysis for Chinese population

**DOI:** 10.18632/oncotarget.18548

**Published:** 2017-06-16

**Authors:** Tao Xiao, Lei Fu, Zhigang Jie

**Affiliations:** ^1^ Department of Gastrointestinal Surgery, First Affiliated Hospital, Nanchang University, Nanchang, Jiangxi 330006, China; ^2^ Department of Stomatology, Third Affiliated Hospital, Nanchang University, Nanchang, Jiangxi 330006, China

**Keywords:** SATB1, gastrointestinal neoplasm, meta-analysis, invasion, metastasis

## Abstract

**Background:**

Gastrointestinal neoplasm (GIN) is the most common neoplasm in China. The global chromatin organizer SATB1 (special AT-rich sequence binding protein 1) is aberrantly expressed in multiple human neoplasms. We conducted this meta-analysis to investigate whether the invasion and metastasis of GIN correlates with SATB1 levels in tumor tissues in Chinese patients.

**Materials and Methods:**

Eligible studies were identified through multiple search strategies in the databases PubMed, Embase, Medline, CNKI, and WANFANG, and the relevant clinicopathological data were extracted. Data were pooled using the Mantel-Haenszel fixed-effects or DerSimonian-Laid random-effects model.

**Results:**

Fourteen studies consisting of 1622 patients were included. There were 3, 3, and 8 studies that evaluated esophageal, gastric, and colorectal cancers, respectively. The overall mean percentage of patients with elevated SATB1 levels was 47.84%. Among patients with GIN, SATB1 overexpression was associated with depth of invasion (T stage: RR 1.27, 95% CI 1.18–1.36, *P* = 0.000), regional lymph node metastasis (N stage: RR 1.51, 95% CI 1.22–1.87, *P* = 0.000), and distant metastasis (M stage: RR 2.54, 95% CI 1.46–4.41, *P* = 0.001). The tumor type most closely linked with invasion and metastasis in GIN was gastric cancer (RR for T stage: 1.64, RR for N stage: 1.68, RR for M stage: 3.15).

**Conclusions:**

invasion and metastasis of GIN in Chinese patients correlates with SATB1 overexpression in tumor tissues, most profoundly in gastric cancer.

## INTRODUCTION

Gastrointestinal neoplasm (GIN) is the most common type of neoplasm in China. According to the annual report of the Chinese Cancer Registry published in 2015, the proportion of GIN in new cases of cancer is 30.31%. Mortality due to GIN is 31.53% of the overall mortality associated with human neoplasms [1]. Although the incidence and mortality rates of esophageal and stomach malignancy are slowly declining, GIN remains an important public health issue in China. By the time GIN is suspected and diagnosed in Chinese patients, the cancer is usually well advanced [2], and prognosis is poor due to the cancer's vigorous invasive and metastatic abilities. The identification of novel biomarkers for early diagnosis is vital to improving patient outcomes.

SATB1 (special AT-rich-binding protein 1) is a global genome organizer that can change chromatin architecture to reprogram gene expression profiles of the genome. SATB1 provides a nuclear architectural platform that anchors hundreds of genes, regulating gene expression by interacting with specific genomic sequences [3]. The function of SATB1 in promoting cancer progression and metastasis was reported for the first time in breast cancer [4]. SATB1 is reportedly overexpressed in GIN, and SATB1 overexpression significantly correlates with tumor invasion and metastasis, as determined by TNM classification [5, 6]. SATB1 also promotes aggressive tumor behavior in several types of neoplasms, including gastric and colorectal cancers [7]. Many studies conducted on GIN had small sample sizes, and some of the results on SATB1's role in GIN were not consistent; for instance, in some studies, high levels of SATB1 were not correlated with shorter survival [8]. The specific role of SATB1 in GIN remains controversial because of the small sample sizes and inconsistent of most of the relevant studies [8, 9].

We conducted the present meta-analysis to investigate the clinical significance of SATB1 for predicting the status of invasion and metastasis of GIN in Chinese patients.

## MATERIALS AND METHODS

### Selection of eligible studies

**Figure 1:**
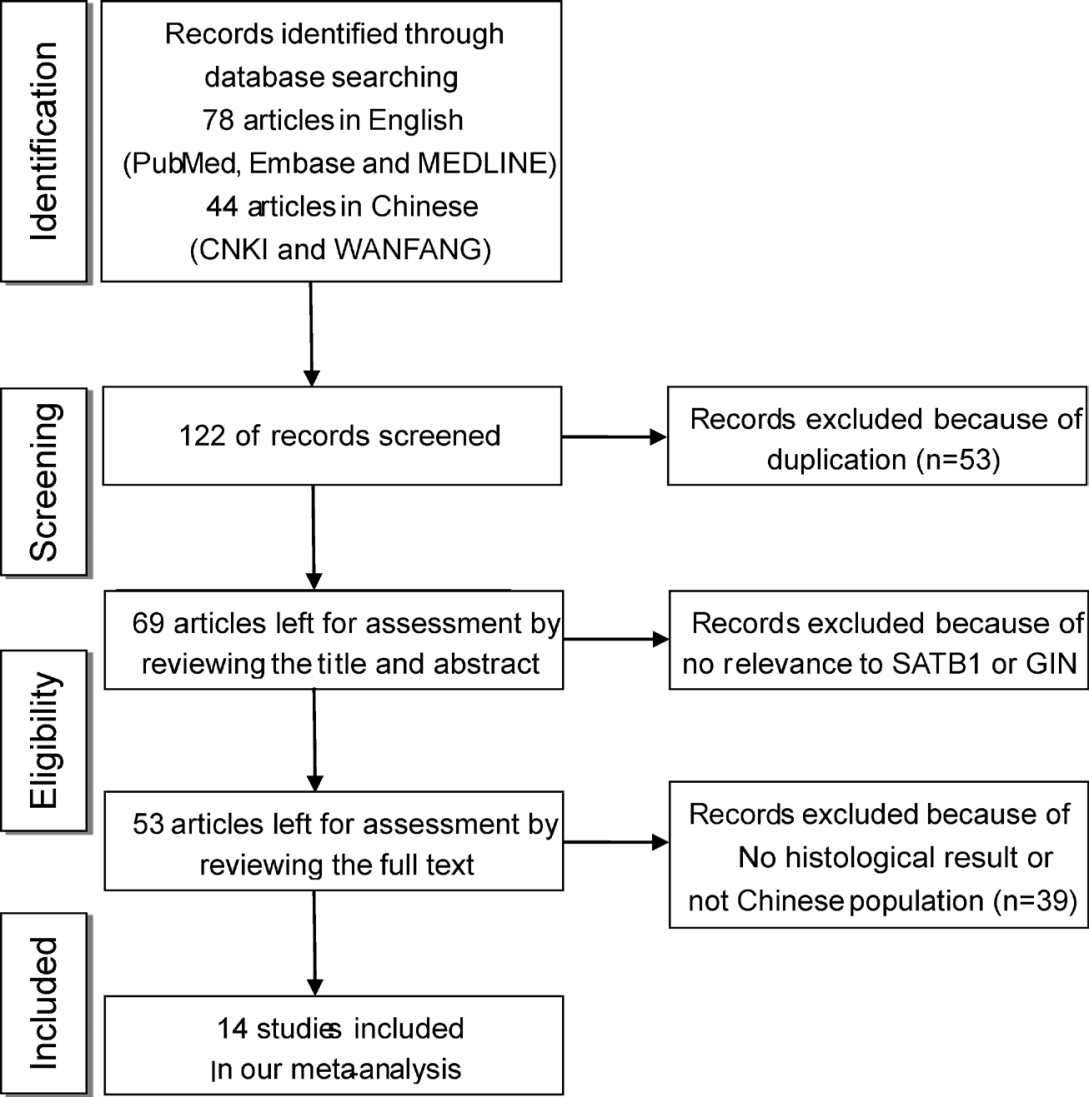
Flow chart summarizing the literature search and selection of eligible studies

Our meta-analysis was performed based on the PRISMA statement (Preferred Reporting Items for Systematic Reviews and Meta-Analyses) [10] (Figure 1). We searched the databases PubMed, Embase, Medline, CNKI (China National Knowledge Infrastructure) and Wanfang for studies concerning SATB1 in GIN published from January 1, 1945 to December 1, 2015. The medical terms “SATB1 OR special AT-rich binding protein 1” and “gastrointestinal neoplasms” were used, and the results were limited to human studies. In addition, we used the entry “SATB1 OR special AT-rich binding protein 1” and the name of each specific tumor (esophageal neoplasm, intestinal neoplasm, and stomach neoplasm) to find additional studies. Citation lists of retrieved articles were manually screened to ensure the sensitivity of the search strategy. Initially, we identified 78 entries published in English and 44 entries published in Chinese.

The selected studies met the following inclusion criteria: SATB1 levels were measured and could be differentiated as elevated or normal, the diagnosis of GIN was confirmed by histopathology examination, clinical characteristics and pathological results were provided or could be extracted from the published data, and the population studied was Chinese (Figure 1). Excluded studies were case reports, letters, or reviews without original data or original studies that did not meet all of the inclusion criteria. For studies with the same or overlapping data reported by the same authors, we selected the study that contained the most details.

### Data collection and process

Two authors (Tao Xiao and Lei Fu) independently collected information using predefined data extraction forms. For conflicting evaluations, an agreement was reached after discussion with the third investigator (Zhigang Jie). Data were extracted from the included studies, including the first author's name, year of publication, language of publication, total number of patients, tumor type, depth of invasion, lymph node and distant metastases, and level of SATB1 expression (Table 1).

**Table 1:** Percentage of patients with SATB1 overexpression reported in the selected studies

					SATB1, *n* (%)		
	Year	Language	Cases, *n*	Tumor type	Overexpression	Normal expression	Source of SATB1 antibody
Cheng C [5]	2010	English	102	Stomach	48.04% (49)	51.96% (53)	Abcam Inc., USA
Lu XM [20]	2010	English	118	Stomach	47.46% (56)	52.54% (62)	Abcam Inc., USA
Meng WJ [21]	2011	English	93	Rectal	44.09% (41)	55.91% (52)	Epitomics, USA
Zhang J [25]	2012	English	80	Colorectal	58.75% (47)	41.25% (33)	BD Biosciences, USA
Zhang Y [6]	2014	English	520	Colorectal	39.42% (205)	60.58% (315)	Sigma-Aldrich, USA
Niu YF [41]	2015	English	131	Colorectal	48.85% (64)	51.15% (67)	Abcam Inc., USA
Cong QX [26]	2015	English	180	Esophageal	48.33% (87)	51.67% (93)	Abcam Inc., USA
Chen CM [42]	2012	Chinese	58	Colorectal	46.55% (27)	53.45% (31)	BD Biosciences, USA
Gao C [27]	2013	Chinese	39	Colorectal	69.23% (27)	30.77% (12)	Abcam Inc., USA
Liu YH [28]	2013	Chinese	50	Colorectal	82.00% (41)	18.00% (9)	Santa Cruz, China
Yang H [29]	2013	Chinese	30	Stomach	66.67% (20)	33.33% (10)	Epitomics, USA
Wang LJ [31]	2013	Chinese	93	Esophageal	44.09% (41)	55.91% (52)	Epitomics, USA
Du C [43]	2014	Chinese	80	Rectal	46.25% (37)	53.75% (43)	Null information
Liu K [23]	2015	Chinese	48	Esophageal	70.83% (34)	29.17% (14)	Bioss, China

### Statistical analysis

Statistical analyses were conducted using STATA 12.0 statistical software (Stata, USA). To assess associations between SATB1 expression level and clinico-pathological features in Chinese patients with GIN, relative risk (RR) and 95% confidence intervals (95% CI) were calculated using the Mantel-Haenszel fixed-effects or Der Simonian-Laird random effects model [11, 12]. The statistical significance of pooled RRs was estimated using a *Z*-test. We used Cochran's *Q*-test and the *I*^2^ test to assess heterogeneity between the studies, and *P* < 0.05 was considered statistically significant [13, 14]. The random effects model was applied when there was evidence of significant heterogeneity (*P* > 0.10 or *I*^2^ test < 50%). Otherwise, the fixed effects model was used [15, 16].

Univariate and multivariate meta-regression analyses were applied to assess the potential sources of heterogeneity, and subgroup analyses were conducted to identify the source of heterogeneity [17]. Sensitivity analyses were applied after the sequential removal of each study to evaluate the influence of single studies on the overall estimate [13]. The effect of publication bias was detected by funnel plot. In case of funnel plot asymmetry, the trim-and-fill method was used to distinguish publication bias from other causes of asymmetry [18, 19]. All statistical tests were two-sided.

## RESULTS

We identified 14 studies that assessed SATB1 expression in 1,622 Chinese patients with GIN (Table 1). The median sample size was 87. The years of publication ranged from 2010 to 2015. Seven articles were published in English, and the rest were written in Chinese.

### Correlation of SATB1 expression with invasion and metastasis of GIN

Except for one study by Lu et al. [20], the remaining studies evaluated the expression of SATB1 in the nuclei of tumor tissues using immunohistochemistry and found that SATB1 expression in tumor tissues was significantly higher than in the corresponding normal tissues [5, 21]. Notably, SATB1 protein expression in tumor nuclei needs to be carefully evaluated to assess its correlation with tumor characteristics and not whole mRNA levels due to SATB1 expression in stromal cells (e.g., lymphocytes) as well [22]. However, Lu et al. [20] reported that in gastric cancer, SATB1 mRNA levels positively correlated with SATB1 proteins as detected by immunohistochemistry.

The overall mean level of SATB1 overexpression was 47.84%, ranging from 39.42% to 82.00%. The mean levels in esophageal, gastric, and colorectal cancer tissues were 54.42%, 54.05%, and 54.39%, respectively.

### Association of SATB1 with the depth of invasion of the primary tumor (T)

**Figure 2:**
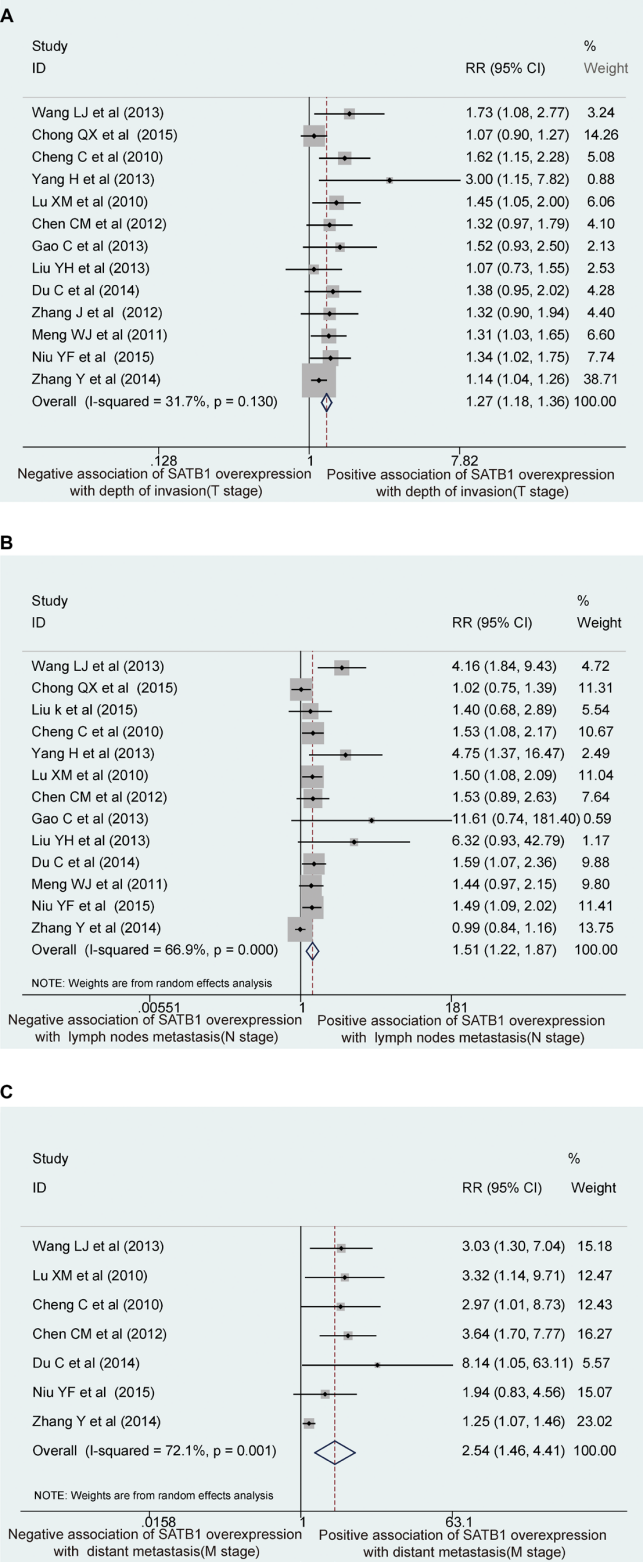
Forest plots of the correlation of SATB1 overexpression with depth of tumor invasion in surrounding tissues (**A**), with regional lymph nodes metastasis (**B**) and with distant metastasis of GIN (**C**). RR, relative risk; CI, confidence intervals.

Thirteen studies, including 1,556 patients, were eligible for the final analysis. Two studies evaluated esophageal cancer, 3 evaluated gastric cancer, and 8 evaluated colorectal cancer. Liu's study was excluded due to the absence of T stage data [23]. There was no heterogeneity across the studies (I^2^ = 31.7%, *P* = 0.13), and the fixed effects model was used. Compared to patients of GIN with normal levels of SATB1, those with overexpressed SATB1 in primary tissue were more likely to have a greater depth of invasion (RR 1.27, 95% CI 1.18–1.36, *P* = 0.000; Figure 2).

Because the funnel plot was asymmetrical, the trim-and-fill method was used to analyze publication bias [24]. Six studies were trimmed and filled, and the adjusted combined effect size was insignificant (pre-trimmed: pooled estimate 0.199, 95% CI 0.133–0.265; post-filled: pooled estimate 0.156, 95% CI 0.095–0.217; Supplementary Figure 1A).

### Association of SATB1 with regional lymph node metastasis (N)

There were 13 studies with 1,556 patients eligible for the final analysis, including 2, 3, and 8 studies regarding esophageal, gastric, and colorectal cancers, respectively. The study by Zhang et al. was excluded due to the absence of N stage data [25]. Heterogeneity existed across the studies (N stage: I^2^ = 66.9%, *P* = 0.00), which remained after the random effects model was applied (N stage: I^2^ = 66.9%, *P* = 0.00, Tau^2^ = 0.08; Figure 2). Therefore, we performed a meta-regression to quantify heterogeneity according to the covariates tumor type and sample size.

**Figure 3:**
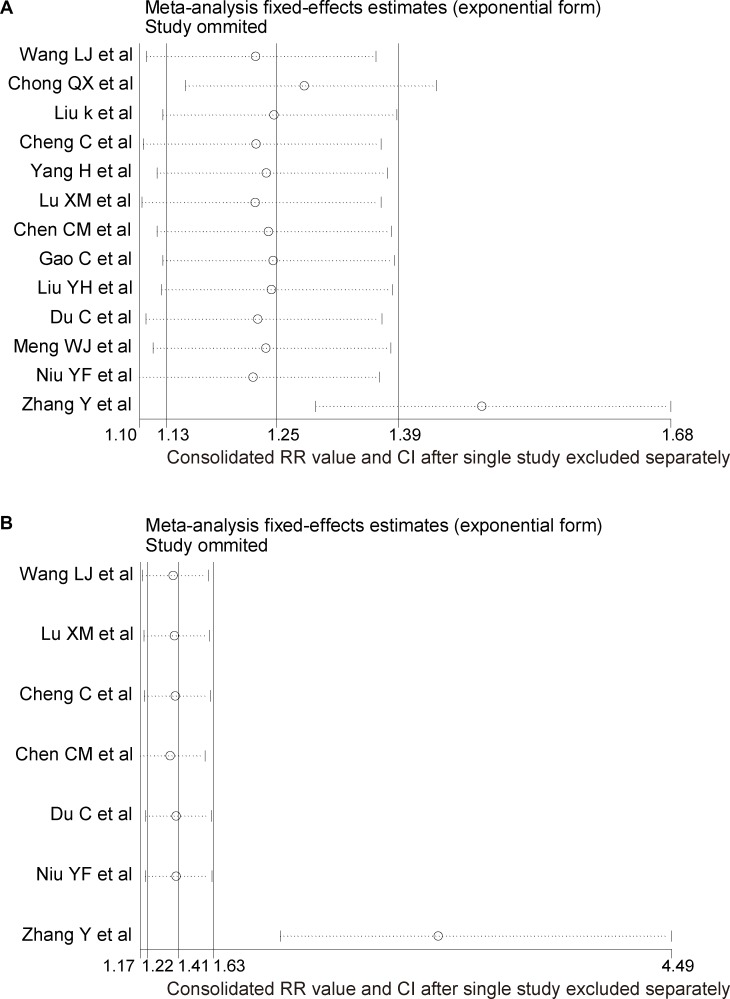
Sensitivity analysis for the studies reporting the association of SATB1 overexpression with regional lymph node metastasis (**A**) and with distant metastasis (**B**). Results were computed by omitting each study in turn. Meta-analysis random-effects estimates (exponential form) were used. The two ends of the dotted lines represent the 95% confidence intervals (CI).

The multivariate meta-regression and univariate meta-regression analyses indicated that sample size was the primary source of heterogeneity (*P* = 0.007, Tau^2^ [multivariate] = 0, Tau^2^ [univariate] = 0.001). The sensitivity analysis found that the study by Zhang et al. [6] was the main source of heterogeneity (Figure 3), and omitting this study did not influence the result (RR 1.58, 95% CI 1.29–1.93, *P* = 0.000 cf. RR 1.51, 95% CI 1.22–1.87, *P* = 0.000; Figure 2). The analysis revealed that regional lymph node metastasis was associated with SATB1 overexpression. The funnel plot was asymmetrical, and the trim-and-fill approach showed that 5 studies should be trimmed and filled. The adjusted combined effect size was insignificant (pre-trimmed: pooled estimate 0.406, 95% CI 0.196–0.616; post-filled: pooled estimate 0.27, 95% CI 0.058–0.482; Supplementary Figure 1B).

### Association of SATB1 with distant metastasis (M)

Eligible for the final analysis were 1, 2, and 4 studies concerning esophageal, gastric, and colorectal cancers, respectively, comprising 1,084 patients. Because of the absence of M stage data, 7 studies were excluded [21, 23, 25–29]. Considering the heterogeneity across studies (M stage: I^2^ = 72.1%, *P* = 0.001), heterogeneity remained after the random effects model was applied (M stage: I^2^ = 72.1%, *P* = 0.001, Tau^2^ = 0.34; Figure 2). Because there were fewer than 10 studies, the meta-regression was not applicable to explore sources of heterogeneity. The sensitivity analysis showed that the study by Zhang et al. [6] was the primary source of heterogeneity (Figure 3). Discarding Zhang et al.'s [6] study removed the heterogeneity (I^2^ = 0.0%, *P* = 0.82), but the conclusion that patients of GIN with SATB1 overexpression suffered more distant metastasis than patients with normal expression was consistent (M stage: RR = 2.54, 95% CI = 1.46 to 4.41, *P* = 0.001 cf. RR = 3.03, 95% CI = 2.05 to 4.49, *P* = 0.000; Figure 2). The funnel plot was asymmetrical. The trim-and-fill method showed that 2 studies should be trimmed and filled, and the adjusted combined effect size was insignificant (pre-trimmed: pooled estimate = 0.916, 95% CI = 0.393 to 1.439; post-filled: pooled estimate = 0.73, 95% CI = 0.30 to 1.161; Supplementary Figure 1C).

### Association of SATB1 with different tumor types

**Figure 4:**
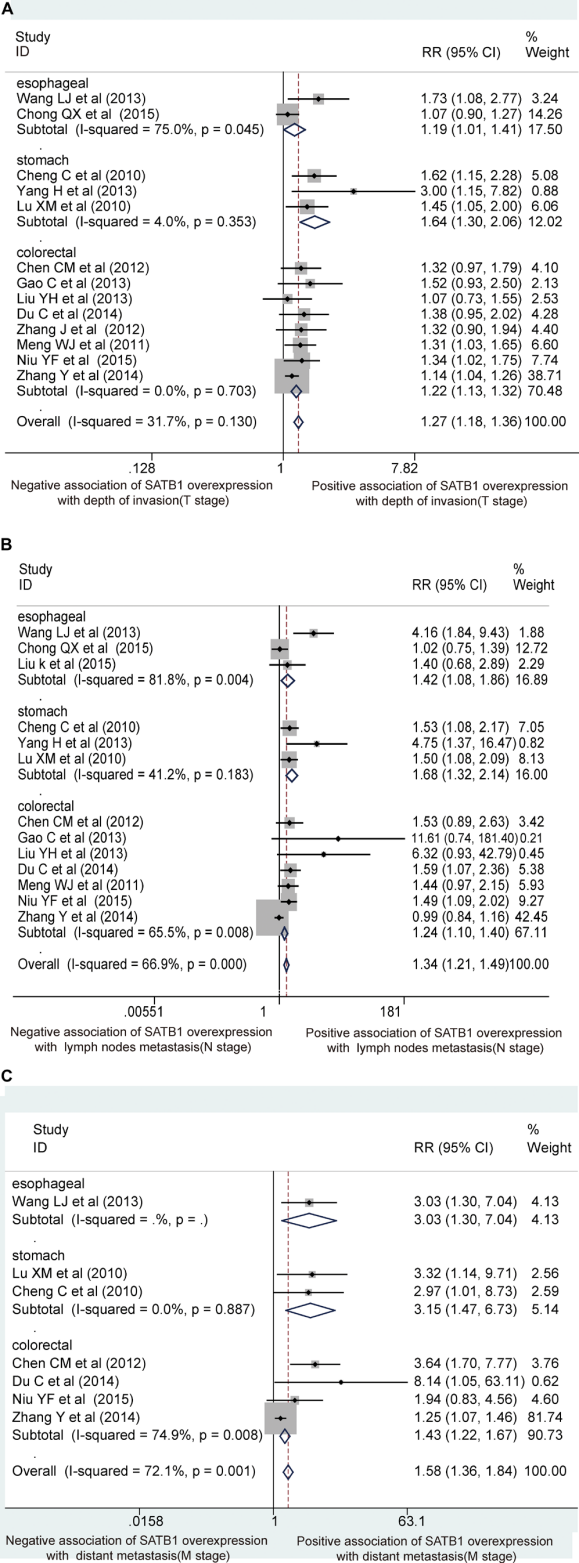
Subgroup analysis of the studies based on the different tumor sites Forest plots depict the association of SATB1 overexpression with depth of invasion (**A**) with regional lymph node metastasis (**B**) and with distant metastasis (**C**)

In the identified studies, 3 evaluated esophageal cancer (*n* = 321), 3 evaluated gastric cancer (*n* = 250), and 8 evaluated colorectal cancer (*n* = 1,051). The expression levels of SATB1 in esophageal, gastric, and colorectal cancer were 50.47%, 50.00% and 46.53%, respectively. To clarify the tumor-specific association of SATB1 with invasion and metastasis, a subgroup analysis based on tumor type indicated that SATB1 overexpression in gastric cancer was most closely linked with invasion and metastasis (RR-T stage: gastric 1.64, colorectal 1.23, esophageal 1.19; RR-N stage: gastric 1.68, esophageal 1.42, colorectal 1.24; RR-M stage: gastric 3.15, esophageal 3.03, colorectal 1.43; Figure 4).

## DISCUSSION

Cancer is a major cause of morbidity and mortality worldwide, and GIN is the most common human cancer [30]. The estimated incidence and mortality rates of Chinese patients with GIN are worse, approximately 30.31% and 31.53%, respectively [1]. Since SATB1 was first found to promote tumor growth and metastasis of breast malignancies in 2008 [4], research has shown that SATB1 may influence tumor prognosis in multiple tumors [3, 7]. In 2010, SATB1 was reported for the first time in Chinese patients with GIN by Cheng et al. [5], who implied that SATB1 expression may serve as a marker for predicting the invasion and metastasis of gastric cancer. Meng et al. in 2011 and Zhang et al. in 2012 reported that SATB1 was overexpressed in colorectal cancer tissues and was significantly involved in tumor progression and infiltration [21, 25]. Wang [31] indicated that SATB1 could significantly promote cell migration and invasion in esophageal squamous cell carcinoma. The function of SATB1 is also tumor-specific [32–35], and multiple effects were observed in different cancer types. Previous studies indicated that SATB1 may enhance the aggressive behavior of GIN. However, most research studies had small sample sizes, different tumor types were investigated, and the results were not consistent [8]. There are several reasons for the inconsistent results. The antagonistic effects of SATB1 and SATB2 in GIN represent one of the primary reasons [36–38]. SATB2 had potential influence on the use of SATB1 as a prognostic marker [39]; however, some studies failed to take SATB2 expression into consideration. Secondly, the small sample sizes of some studies [8] may increase the sampling error and distort the distribution, which could result in unreliable conclusions that are inconsistent with others. Additionally, inconsistent observations about European patients [8] and Australian patients [9] suggest that race may make a difference in SATB1 expression and its relationship with the progression of GIN. Thus, we wanted to avoid this factor and focus our meta-analysis on Chinese patients.

Our present investigation showed that SATB1 overexpression in tissues of GIN ranged from 39.42% to 82.00%, and the median level was 47.84% compared with low or negative expression in normal tissues. The median expression levels of SATB1 in esophageal, gastric, and colorectal cancer were 50.47%, 50.00%, and 46.53%, respectively. Our results indicated that SATB1 overexpression was accompanied by progression of GIN and that the expression level differed according to the tumor type. Although heterogeneity was present across the studies, after clarification and assessment of the heterogeneity source by statistical methods, we can conclude that SATB1 overexpression was associated with depth of invasion, regional lymph nodes, and distant metastasis. The potential molecular mechanism of SATB1 in GIN should be explored further. Interestingly, our subgroup analysis based on tumor type revealed that gastric cancer with invasion and metastasis was the type most closely linked with SATB1 overexpression. This suggests that SATB1 may be a good marker for monitoring gastric cancer. However, considering that the overall sample size of gastric cancer was only 250 cases, a large-scale study should be conducted to verify this conclusion.

Our meta-analysis quantitatively assessed the correlation between SATB1 overexpression and invasion and metastasis of GIN and is more credible than the results reported in previous individual studies. Furthermore, our research is the first to show that gastric cancer is the tumor type most closely linked with SATB1 overexpression. We also found that the detection of SATB1 expression in tumor tissues obtained through endoscopic biopsy may be used to evaluate the invasiveness of the tumor. This could be combined with imaging and other biochemical tests to help surgeons predict tumor metastasis and make clinical decisions. Our results suggest that SATB1 may be a useful prognostic biomarker for clinical outcomes and a novel therapeutic target for treating GIN, which was also suggested by previous investigators [4, 40]. This meta-analysis is limited because it is a literature-based analysis and because the detection methods for SATB1 were not consistent among the different studies. In addition, only 3, 3, and 8 studies that evaluated esophageal, gastric, and colorectal cancers, respectively, were analyzed. More studies are required to validate the conclusion.

In summary, our results indicate that SATB1 overexpression in GIN is associated with higher depth of invasion and presence of nodal and distant metastasis. The influence of SATB1 overexpression is more profound in gastric cancer patients. The detection of SATB1 overexpression in clinical practice may help in predicting the stage of the disease and aid in clinical decision-making.

## SUPPLEMENTARY MATERIALS FIGURES


