# Supplementary material for: SATB1 overexpression correlates with gastrointestinal neoplasms invasion and metastasis: a meta-analysis for Chinese population
Source: Oncotarget. 2017 Jun 16;8(29):48282–90. doi: 10.18632/oncotarget.18548 (PMC5564646; doi:10.18632/oncotarget.18548)
Supplement: Supplementary file 1 [file oncotarget-08-48282-s001.pdf]

SATB1 overexpression correlates with gastrointestinal neoplasms invasion and metastasis: a meta-analysis for Chinese population

SUPPLEMENTARY MATERIALS

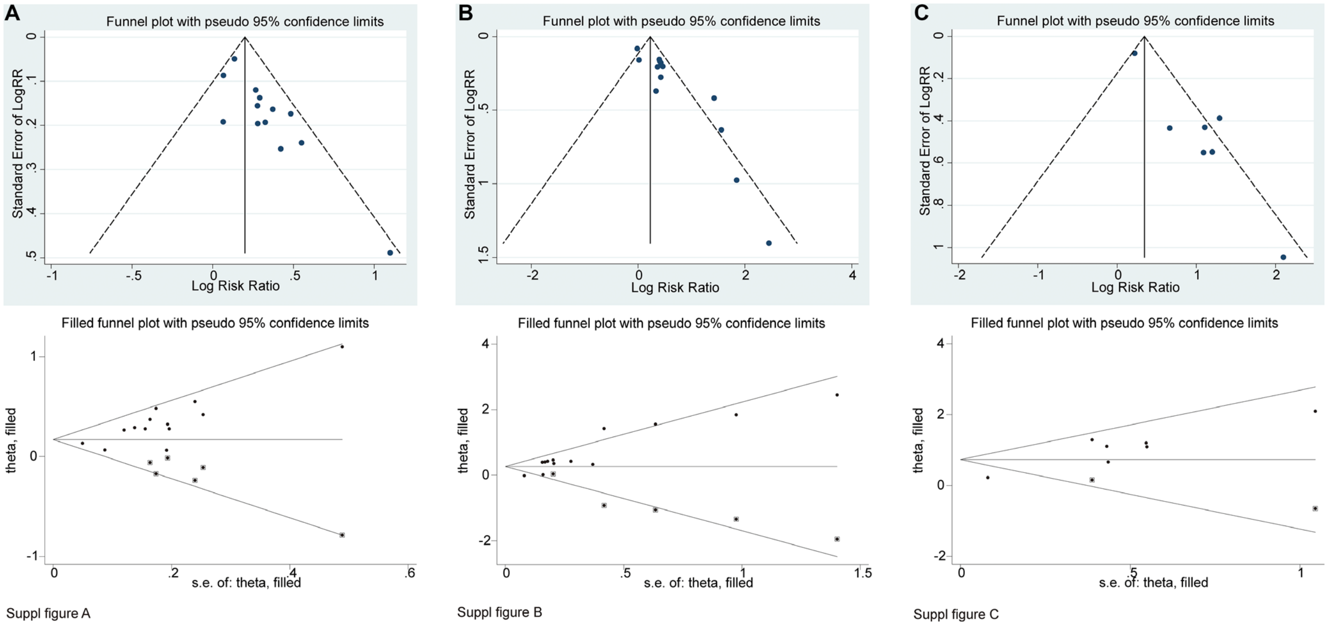

Supplementary Figure 1: Detection and discrimination of publication bias of the included studies for the association of SATB1 with depth of invasion (**A**) with regional lymph node metastasis (**B**) and with distant metastasis (**C**). Each point represents a separate study for the indicated association. Horizontal line represents the mean magnitude of the association.
